# HOME HEALTHCARE ACCESS AND UTILIZATION AMONG VULNERABLE POPULATIONS IN THE US AND PUERTO RICO

**DOI:** 10.1093/geroni/igac059.1541

**Published:** 2022-12-20

**Authors:** Maricruz Rivera-Hernandez

## Abstract

Home health (HH) care utilization among Medicare beneficiaries has exploded, serving over 3.3 million users (The Medicare Payment Advisory Commission, 2021). With the aging of the population and the preference for Home- and Community-Based Services and/or person-centered care delivered in the home, HH care use is expected to continue increasing. Prior research has highlighted inequitable access and use of HH rooted in structural and social determinants of health (Fashaw-Walters et al., 2022). However, more research is needed about disparities in timely access to quality of care and outcomes among racial and ethnic and rural populations. Furthermore, the literature regarding post-acute care has often excluded Puerto Rico HH beneficiaries (Rivera-Hernandez et al., 2020). Given the need to ensure equity of care post-pandemic, there is a pressing need to understand disparities in care and outcomes, specifically using representative data. This symposium will feature four presentations that provide novel insight regarding HH care utilization and outcomes among vulnerable populations. Individual presentations will describe 1) Home health care services utilization among Medicare beneficiaries in Puerto Rico; 2) Public reporting role in exacerbating disparities in access; 3) Differences in home healthcare latency following hospitalization for ADRD patients by race/ethnicity and rural and urban locations; 4) Racial disparity in the start of home healthcare in high-risk ADRD patients by the quality of home health and impact on rehospitalization. In addition, studies in this panel will discuss policy and clinical implications, as well as directions for future research regarding equitable access and health outcomes among HH users.

